# Recurrence quantification analysis of gait for computer-aided diagnosis of Parkinson's disease

**DOI:** 10.3389/fnagi.2026.1791253

**Published:** 2026-05-21

**Authors:** Huan Zhao, Liangyuan Li, Junxiao Xie, Junyi Cao, Qiumin Qu, Wei-Hsin Liao, Yongning Deng

**Affiliations:** 1 Department of Neurology, The First Affiliated Hospital of Xi'an Jiaotong University, Xi'an, China; 2 School of Mechanical Engineering, Xi'an Jiaotong University, Xi'an, China; 3 Department of Mechanical and Automation Engineering, The Chinese University of Hong Kong, Shatin, Hong Kong SAR, China

**Keywords:** entropy features, intelligent diagnosis, nonlinear dynamics, recurrence plot, recurrence quantification analysis

## Abstract

**Introduction:**

Parkinson's disease (PD) is a typical neurodegenerative disorder characterized by progressive motor impairments. Gait analysis offers a promising avenue for non-invasive PD diagnosis, yet extracting discriminative features from gait signals remains challenging.

**Methods:**

This study proposed an intelligent diagnostic framework for PD based on quantitative analysis of recurrence plots derived from plantar pressure gait signals. Gait data were collected from 61 PD patients and 48 healthy subjects using a wearable gait acquisition system. Various recurrence plots representations, including thresholded, non-thresholded, basic, cross, and joint recurrence plots were constructed. From these recurrence plots, traditional recurrence quantification analysis (RQA) features and novel recurrence plot entropy features derived from compressed one-dimensional sequences of non-thresholded recurrence plots were extracted.

**Results:**

Statistical analysis revealed that recurrence plot entropy features particularly from cross recurrence plots exhibited superior discriminability. Pressure signals from the heel and toe positions showed the highest specificity. For classification, an integrated feature set combining temporal, pressure, and recurrence plot features achieved the best diagnostic performance using a Cubic Support Vector Machine (CSVM) model, yielding a maximum accuracy of 92.71% in distinguishing PD patients from healthy controls (HC).

**Discussion:**

The results demonstrated that the proposed quantitative recurrence plots analysis framework provides a highly effective and automated approach for intelligent PD diagnosis based on gait dynamics.

## 1 Introduction

Parkinson's disease (PD) is a common neurodegenerative disorder that predominantly affects the elderly population. Its incidence increases markedly with advancing age (Zhang et al., 2005; Chen et al., 2015). PD affects multiple physiological systems and introduces irregular, nonlinear fluctuations in their outputs, growing attention has been directed toward dynamical-systems-based methods to better understand these multifaceted alterations in PD. Recurrence plots have been widely used to mine the hidden information contained in time series and have played an important role in elucidating the mechanisms of PD. Flood et al. (2019) investigated differences in surface Electromyography features and found that PD patients exhibited significantly higher determinism. Shinoda et al. (2021) employed Poincaré mapping and recurrence quantification analysis (RQA) to identify randomness and chaotic dynamics in Electrocardiograph signals from PD animal models. Rodriguez-Sabate et al. (2021) analyzed blood-oxygen-level-dependent signals in healthy controls (HC) and PD patients using binary recurrence plots and binary cross-recurrence plots, uncovering complex dynamic interactions between brain regions and PD-related alterations. Pham (2021) used the maximum recurrence eigenvalue derived from convolutional fuzzy recurrence plots to discriminate behavioral patterns between wild-type and mutant *C. elegans*, as well as gait patterns between healthy controls (HC) and PD patients.

Features extracted from recurrence plots have demonstrated outstanding performance in distinguishing PD patients from healthy individuals. Serrano-Reyes et al. (2022) employed multiscale recurrence analysis and found increased determinism and reduced state transitions in PD cortical circuits, consistent with clinical rigidity and bradykinesia. Pham (2018a) applied recurrence-based methods to keystroke timing series to characterize and classify healthy controls and early-stage PD subjects. Pham and Oyama-Higa (2018) applied nonlinear dynamical analyses to wearable photoplethysmography signals and achieved the best classification performance using fuzzy recurrence-based features. Sasidharan et al. (2025) constructed recurrence and cross-recurrence plots from a public Electroencephalograph dataset and used these features in machine-learning classifiers, achieving strong specificity and overall classification performance. Murugappan et al. (2020) used RQA features from recurrence plots to classify HC and PD, achieving average accuracies of 89.17 and 84.50%, respectively.

Recurrence plot of signals can directly be inputted to deep learning models to automatically extract PD-related features. Afonso et al. (2019) converted handwriting kinematic data into recurrence plot images and used CNNs for PD identification, achieving accuracies above 87%. Cantürk (2021b) transformed time-series signals into fuzzy recurrence plot images and evaluated PD classification performance using deep learning. Pham et al. (2019) used fuzzy recurrence plots with LSTM networks to distinguish HC from early-stage PD patients. Skaramagkas et al. (2023) used speech recurrence plots to distinguish PD patients from HCs, achieving 87% accuracy. Fujita et al. (2021) proposed a novel hybrid RNN-CNN architecture for PD detection using recurrence-plot representations of speech signals. Xu et al. (2024) developed a CNN-based diagnostic framework for PD in which temporal measurement data were transformed into recurrence-based activity maps for network training, achieving an accuracy of 84.52%. However, these deep learning approaches lack transparency and interpretability.

Gait analysis has emerged as a promising non-invasive tool for PD assessment, and several studies have applied recurrence plots to gait signals. Schmit et al. (2006) used RQA on postural sway and found PD patients had greater center-of-pressure variability and pronounced temporal dynamics than healthy controls. Pham (2022) applied tensor decomposition of fuzzy recurrence plots from stride-interval signals to analyze gait dynamics, revealing distinct patterns across young adults, older adults, and PD patients. Yu et al. (2017) proposed a method combining recurrence plots and RQA to quantify the complexity of nonlinear gait time series, enabling effective classification of individuals by age and PD status. Afsar et al. (2018) used RQA on gait force spectra and found entropy, determinism, and mean diagonal line length varied with PD severity. Geman (2011) used recurrence and chaos-analysis techniques on PD gait signals to evaluate nonlinear parameters and suggested early screening. Pham (2018b) converted gait time series into fuzzy recurrence plot images and extracted texture features for classification, effectively distinguishing PD patients from controls. Cantürk (2021a) transformed PD gait signals into fuzzy recurrence plots, extracted deep features, and achieved classification accuracies of 1.00 and 0.99 for female and male patients, respectively.

Despite the progress made in diagnosing PD using gait-analysis methods, several challenges remain when considering the complexity of PD progression, the substantial inter-individual variability, and the eventual goal of integrating research findings into real clinical practice to support medical experts. Current gait-feature studies still face major limitations in identifying specific biomarkers, enabling accurate early diagnosis and assessment, and translating research outcomes into effective clinical interventions. The extraction of nonlinear information from gait signals remains superficial, and objective gait biomarkers capable of clearly distinguishing PD from other neurological disorders are still lacking. To address this issue, this study proposes an intelligent diagnostic framework for PD gait analysis. Considering the unique characteristics of PD gait signals, the method converts gait data into two-dimensional images using recurrence plots.

## 2 Method

### 2.1 Phase space reconstruction

Recurrence plot is a visualization to transform hidden recurrence patterns in time series into visual images, revealing the similarity, recurrence, and dynamical characteristics within time series. Takens' embedding theorem (Noakes, 1991) was proposed to address the problem of high-dimensional phase space reconstruction. Since the reconstructed space is topologically equivalent to the original dynamical system, all analysis and prediction tasks on the original sequence can be transferred to this reconstructed space.

For a time series of length *N*, *x*(*n*),*n* = 1, 2, ..., *N*, it can be transformed into an *m*-dimensional vector through phase space reconstruction, with the specific calculation formula shown in Equation 1:


$$\begin{array}{l} X_{m}\left(n\right)=x\left(n\right),x\left(n+\tau \right),...,x\left(n+\left(m-1\right)\tau \right) \end{array}$$ (1)

where, *i* = 1, 2, ..., *N* – (*m* – 1)τ*, m* is the embedding dimension, τ corresponding to delay time. These two parameters are crucial for determining the quality of phase space reconstruction, and their values are of paramount importance: if *m* is too small, false nearest neighbors will occur, where points that are not adjacent in high dimensions appear adjacent in low dimensions; conversely, if *m* is too large, it unnecessarily increases computational complexity. Similarly, if τ is too small, it leads to lack of independence between phase space vectors; whereas if τ is too large, vectors become completely uncorrelated, rendering the reconstruction meaningless.

#### 2.1.1 Determining delay time

Mutual information originates from physics and is commonly used to quantify the correlation between two events. The delay time using the mutual information method is briefly described as follows: Given two discrete time series *S* = (*s*_1_, *s*_2_, …, *s*_*m*_) and *Q* = (*q*_1_, *q*_2_, ..., *q*_*n*_), first calculate their respective information entropies *H*(*S*) and *H*(*Q*), with calculation formulas shown in Equations 2, 3, to measure their average information content:


$$\begin{array}{l} H\left(S\right)=-\overset{m}{\underset{i=1}{\sum }}Ps\left(s_{i}\right)\;Ps\left(s_{i}\right) \end{array}$$ (2)

$$\begin{array}{l} H\left(Q\right)=-\sum_{j=1}^{n}P_{\varrho }\left(q_{j}\right)\;P_{\varrho }\left(q_{j}\right) \end{array}$$ (3)

where *P*(s_*i*_) is probability of event *s*_*i*_ occurring, *P*(*q*_*j*_) is probability of event *q*_*j*_ occurring.

Subsequently, calculate the joint information entropy *H*(*S, Q*) of the two sequences as shown in Equation 4 to characterize their overall uncertainty:


$$\begin{array}{l} H\left(S,Q\right)=-\overset{m}{\underset{i=1}{\sum }}\overset{n}{\underset{j=1}{\sum }}Ps,\varrho \left(s_{i},q_{j}\right)log_{2}Ps,\varrho \left(s_{i},q_{j}\right) \end{array}$$ (4)

where *P*(P(*x*_*i*_, *y*_*j*_) is joint probability of events *x*_*i*_ and *y*_*j*_ occurring together.

Based on the above entropy values, the mutual information *I*(*S, Q*) between sequences *S* and *Q* is calculated as in Equation 5:


$$\begin{array}{l} I\left(S,Q\right)=H\left(S\right)+H\left(Q\right)-H\left(S,Q\right) \end{array}$$ (5)

To further standardize this value, *I*(*S, Q*) is modified to Equation 6:


$$\begin{array}{l} I\left(S,Q\right)=\frac{I\left(S,Q\right)}{\sqrt{H\left(S\right)\times H\left(Q\right)}} \end{array}$$ (6)

Applying the above theory to gait signal analysis, let *S* = *x*(*n*), *Q* = *x*(*n*+τ), and *n* = 1, 2, …, *N*−τ. Thus, *C* is transformed into a function with τ as the independent variable as shown in Equation 7:


$$\begin{array}{l} I\left(\tau \right)=\frac{I\left(x\left(n\right),x\left(n+\tau \right)\right)}{\sqrt{H\left(x\left(n\right)\right)\times H\left(x\left(n+\tau \right)\right)}} \end{array}$$ (7)

By iteratively calculating *I*(τ) corresponding to different integer values of τ and plotting its function curve, the first local minimum point of this curve is selected as the optimal delay time.

#### 2.1.2 Determining embedding dimension

Currently, most studies use the geometric invariant method and false nearest neighbors method to determine the embedding dimension value. The geometric invariant method determines embedding dimension through intrinsic geometric characteristics of the data, offering advantages such as strong nonlinear adaptability, high automation, and good stability. However, its high computational complexity, sensitivity to data noise, dependence on geometric assumptions, and limited generalizability restrict its applicability. From a geometrical perspective, insufficient phase space dimension causes compression effects on signal trajectories and generates false nearest neighbors. The core idea of the false nearest neighbors method is that by progressively increasing the embedding dimension, these false nearest neighbors arising from dimensionality reduction can be systematically eliminated, thereby gradually approaching and restoring the true dynamical characteristics of the system. This method features intuitive principles and computational efficiency. Its calculation procedure is briefly described as follows:

Let $X_{m}\left(n^{\prime }\right)$ be the nearest neighbor of vector *X*_*m*_(*n*) in *m*-dimensional phase space, defined as in Equations 8, 9:


$$\begin{array}{l} X_{m}\left(n^{\prime }\right)=x\left(n^{\prime }\right),\;x\left(n^{\prime }+\tau \right),...,x\left[n^{\prime }+\left(m-1\right)\tau \right] \end{array}$$ (8)

where *n*′ satisfies:


$$\begin{array}{l} n^{\prime }=1,2,...,N-\left(m-1\right)\tau ,\;and\;n^{\prime }\neq n \end{array}$$ (9)

Accordingly, the Euclidean distance between the two vectors $X_{m}\left(n^{\prime }\right)$ and *X*_*m*_(*n*) can be calculated as shown in Equation 10:


$$\begin{array}{l} Y_{m}\left(n,\;n^{\prime }\right)=||X_{m}\left(n\right),X_{m}\left(n^{\prime }\right)|| \end{array}$$ (10)

When the embedding dimension increases from *m* to *m* + 1, this distance will also change. Let the new distance between the two vectors in (*m* + 1)-dimensional phase space be $Y_{m+1}\left(n,n^{\prime }\right)$, calculated as shown in Equation 11:


$$\begin{array}{l} Y_{m+1}\left(n,n^{\prime }\right)=\sqrt{{\left[Y_{m}\left(n,n^{\prime }\right)\right]}^{2}+{\left[x\left(n+m\tau \right)-x\left(n^{\prime }+m\tau \right)\right]}^{2}} \end{array}$$ (11)

If the difference between $Y_{m+1}\left(n,n^{\prime }\right)$ and $Y_{m}\left(n,n^{\prime }\right)$ is significant, then this point is determined to be a false nearest neighbor generated by projection when the phase space dimension is reduced from *m* + 1 to *m*.

To quantify this determination, introduce the variable *Q*_1_(*m, n*) as shown in Equation 12:


$$\begin{array}{l} Q_{1}\left(m,n\right)=\sqrt{\frac{{\left[x\left(n+m\tau \right)-x\left(n^{\prime }+m\tau \right)\right]}^{2}}{{\left[Y_{m}\left(n,n^{\prime }\right)\right]}^{2}}} \end{array}$$ (12)

Set a threshold *Q*_τ_, if *Q*_1_(*m, n*) > *Q*_τ_, then $Y_{m+1}\left(n,n^{\prime }\right)$ can be confirmed as a false nearest neighbor of $Y_{m}\left(n,n^{\prime }\right)$. The false nearest neighbors method effectively determines the optimal embedding dimension through the above mechanism: as the embedding dimension increases, the proportion of false nearest neighbors gradually decreases. When this proportion falls below 5%, the phase space is considered sufficiently unfolded, and the corresponding *m* value is taken as the desired optimal embedding dimension.

### 2.2 Construction of recurrence plot

Various recurrence plots were analyzed. First, taking a one-dimensional nonlinear time series segment from a single system as an example, thresholded and non-thresholded recurrence plots are introduced based on basic recurrence plots. Then, taking one-dimensional nonlinear time series segments *x*(*n*) and *y*(*n*) from two related systems as examples, cross recurrence plots and joint recurrence plots are introduced based on non-thresholded recurrence plots.

#### 2.2.1 Thresholded recurrence plot

After performing phase space reconstruction on a one-dimensional nonlinear time series, the corresponding set of *m*-dimensional vectors can be obtained. From this set, select two vectors *X*_*m*_(*i*) and *X*_*m*_(*j*) corresponding to different time points *i* and *j*, and calculate the Euclidean distance *r*_*i, j*_ = ||*X*_*m*_(*i*)−*X*_*m*_(*j*)|| between them. Based on this, the matrix expression for the recurrence plot is defined as:


$$\begin{array}{l} R_{i,j}=\theta \left(\varepsilon -r_{i,j}\right) \end{array}$$ (13)

where *i, j* = 1, 2, ..., *N*; ε is the distance threshold, taken as 10% of the phase space diameter; Θ is the Heaviside function, whose mathematical definition is:


$$\begin{array}{l} \theta (x)=\{\begin{array}{ll} 1, & x>0\\ 0, & x\leq 0 \end{array} \end{array}$$ (14)

From Equations 13, 14, it can be seen that *R*_*i, j*_ is a matrix composed of elements 0 and 1, with identical number of rows and columns, both equal to *N*−(*m*−1)τ. This matrix is called the recurrence matrix. In this matrix, the value 0 corresponds to a white point in the final visualization, while the value 1 corresponds to a black point. Through this approach, the recurrence matrix is transformed into a thresholded recurrence plot.

#### 2.2.2 Non-thresholded recurrence plot

During recurrence plot generation, parameter configuration directly affects the morphology of the output image, which may subsequently constrain the efficiency of convolutional neural networks in extracting image classification features, ultimately affecting the overall classification performance of the model. Improper threshold selection leads to loss of critical feature information in the generated recurrence plot, which is crucial for the performance of data-driven deep learning models.

To address this issue, this study employs the non-thresholded recurrence plot method during the data reconstruction phase. The core of this method is that after calculating the distance matrix *r*_*i, j*_ = ||*X*_*m*_(*i*)−*X*_*m*_(*j*)||, it abandons the binarization process of comparing it with the distance threshold, and instead directly visualizes the distance matrix. In this case, each element value in the distance matrix directly corresponds to the color intensity of a pixel point in the recurrence plot. Therefore, non-thresholded recurrence plots no longer generate binary images with only black and white colors, but instead continuously and richly map the point-to-point distance information in high-dimensional phase space into two-dimensional images with different color intensities.

#### 2.2.3 Cross recurrence plot

Compared to basic recurrence plots, cross recurrence plots represent an extension to binary systems. This method analyzes the intrinsic correlation by detecting the moments when similar states appear in two different dynamical systems, and is suitable for exploring the interaction mechanisms between two different time series. Consider two different dynamical systems, reconstruct them in the same *m*-dimensional phase space, obtaining trajectory vectors *x*_*i*_ and *y*_*j*_ respectively. Following the construction method of the recurrence matrix, the recurrence matrix corresponding to the cross recurrence plot is defined as shown in Equation 15:


$$\begin{array}{l} \text{C}\text{R}=\Theta \left(\varepsilon -||x_{i}-y_{j}||\right) \end{array}$$ (15)

where *i* = 1, 2, ..., *N*_1_8281; *j* = 1, 2, ..., *N*_2_. Since the number of trajectory vectors in the two systems may differ, the recurrence matrix ***CR*** is not necessarily a square matrix, and its number of rows and columns may be different. If different results are obtained when separately estimating the embedding dimensions for the two time series, the larger dimension should be selected to ensure that both are compared in the same dimensional phase space.

#### 2.2.4 Joint recurrence plot

When two time series have different physical dimensions or phase space dimensions, directly comparing the similarity of their state vectors lacks physical meaning. Joint recurrence plots separately calculate the recurrence properties of each system in its own phase space, and then detect the time points when both systems simultaneously exhibit recurrent states, i.e., joint recurrence moments. For two different dynamical systems, let their embedding dimensions be *m*_*x*_ and *m*_*y*_ respectively. The recurrence matrix of the joint recurrence plot is defined as shown in Equation 16:


$$\begin{array}{l} JR_{i,j}^{x,y}=\left(\varepsilon_{x}-\Theta ||x_{i}-x_{j}||\right)\times \left(\varepsilon_{y}-\Theta ||y_{i}-y_{j}||\right) \end{array}$$ (16)

where ε_1_, ε_2_ are distance thresholds for recurrence analysis of the two system, *x*_*i*_, *y*_*i*_ extended to multi-system scenarios, where the multivariate joint recurrence matrix can be expressed as Equation 17:


$$\begin{array}{l} JR_{i,j}^{x_{\left(1,...,n\right)}}\left(\varepsilon_{x\left(1\right)},...,\varepsilon_{x\left(1\right)}\right)=\overset{n}{\underset{k=1}{\prod }}R_{i,j}^{x_{\left(k\right)}}\left(\varepsilon_{x\left(k\right)}\right) \end{array}$$ (17)

When the trajectory vector of the first system *x*_*j*_ is within the neighborhood of *x*_*i*_, and simultaneously the trajectory vector of the second system *y*_*j*_ is also within the neighborhood of *y*_*i*_ i.e., the recurrent states of both systems occur simultaneously, then a joint recurrence is occurred at this moment. Compared with cross recurrence plots that focus more on analyzing correlation relationships between different parts of the same system that undergo different physical processes, joint recurrence plots allow each system to maintain an independent phase space, making them more suitable for studying two systems that mutually influence each other while maintaining their respective dynamical characteristics.

#### 2.2.5 Construction of recurrence plots

**Figure 1:**
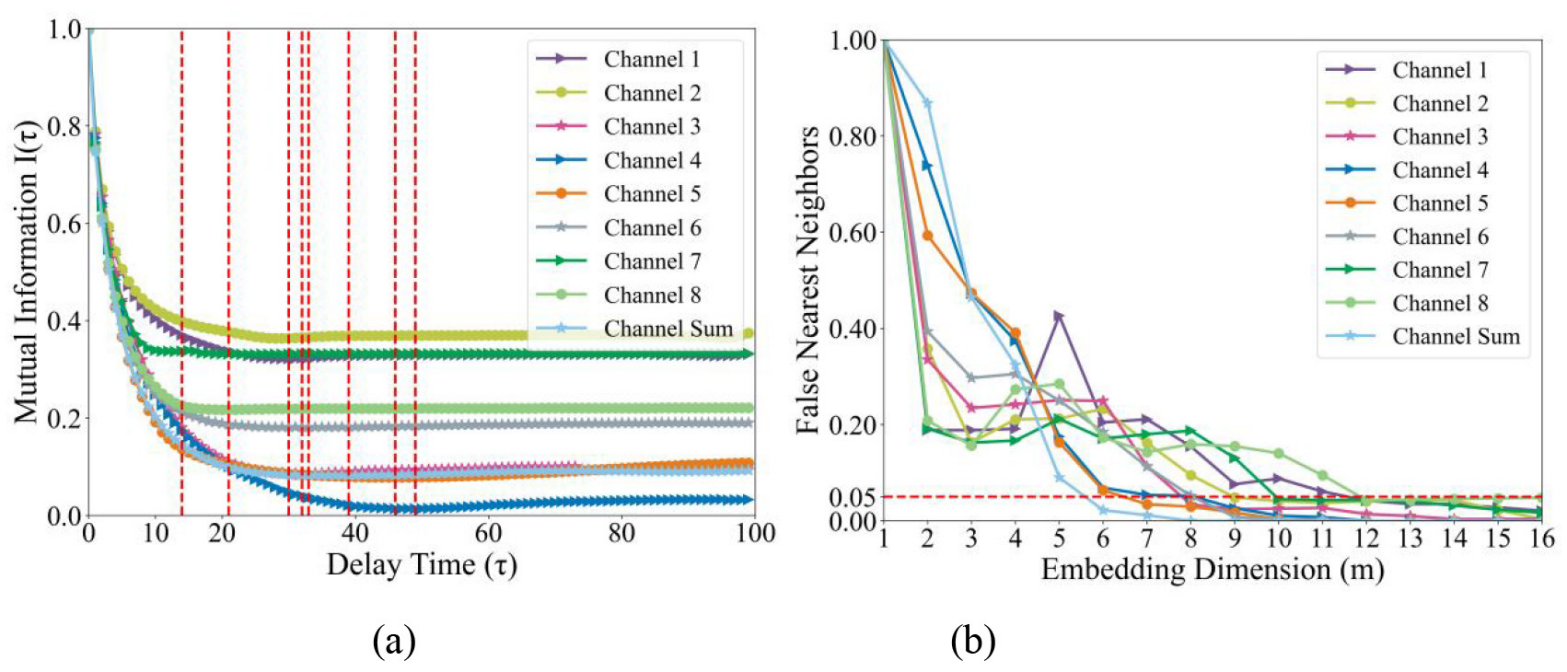
Influence of constructing parameter. **(a)** Mutual information; **(b)** False Nearest Neighbors.

First, the original plantar pressure signals are preprocessed, extracting a one-dimensional time series data segment of 7 s in length from each acquisition channel of each subject. Subsequently, recurrence plots are generated for these data segments. Before generating recurrence plots, appropriate delay time τ and embedding dimension *m* need to be determined for the pressure signals. Considering that the plantar pressure signals acquired from the eight channels may differ in stationarity and nonlinear characteristics, using the same τ and *m* parameters uniformly for all channels would lack rationality. Therefore, this study independently calculates the delay time and embedding dimension for the signals from each of the eight channels before plotting recurrence plots, with specific results shown in Figure 1.

The mutual information was employed to calculate delay time. As shown in Figure 1a, the red dashed line in the figure identifies the abscissa position corresponding to the first local minimum point of the mutual information function, and this value is selected as the delay time for the pressure signal. The embedding dimension is determined through the false nearest neighbors method. In Figure 1b, the red dashed line marks the situation where the proportion of false nearest neighbors to all points in their respective phase spaces is 5%. When the selected embedding dimension makes the proportion of false nearest neighbors fall below 5%, i.e., below the red dashed line, the corresponding abscissa value is selected as the optimal embedding dimension for the pressure signal. The specific calculation results of delay time and embedding dimension for the eight independent channels and the summation channel are shown in Table 1.

**Table 1:** Optimal delay time and embedding dimension values for each channel.

Channel	1	2	3	4	5	6	7	8	Sum
Time delay	30	30	33	49	46	32	14	21	39
Embedding dimension	12	9	8	9	7	9	10	12	6

### 2.3 Feature extraction of recurrence plot

RQA method (Webber and Zbilut, 1994), transforming complex texture and structural features in recurrence plots into a series of quantitative indicators. Gao (2001) extended this method by introducing more descriptive indicators. Commonly used recurrence quantification features mainly include:

(1) Recurrence Rate (RR) is the percentage of recurrence points to the total number of points in the recurrence plot, with the mathematical expression as shown in Equation 18:

$$\begin{array}{l} RR=\frac{1}{N^{2}}\overset{N}{\underset{i=1}{\sum }}\overset{N}{\underset{j=1}{\sum }}R_{i,j} \end{array}$$ (18)

(2) Determinism (DET) is the percentage of recurrence points on 45° diagonal lines to the total number of points as shown in Equation 19:

$$\begin{array}{l} DET=\frac{\sum_{l=l_{\min}}^{N}lP\left(l\right)}{\sum_{i=1}^{N}\sum_{j=1}^{N}R_{i,j}} \end{array}$$ (19)

where *l*_min_ corresponds to minimum diagonal line length, generally taken as 2. *P*(*l*) is probability distribution of diagonal lines of length *l*.

(3) Average Diagonal Line Length (*L*) is the average length of line segments parallel to the main diagonal in the recurrence plot, reflecting the average duration of system state similarity persistence and related to the system's predictable time as calculated in Equation 20.

$$\begin{array}{l} L=\frac{\sum_{l=l_{\min}}^{N}lP\left(l\right)}{\sum_{l=l_{\min}}^{N}P\left(l\right)} \end{array}$$ (20)

(4) Maximum Diagonal Line Length (*L*_max_) is defined as the number of recurrence points contained in the longest diagonal line excluding the main diagonal, calculated as shown in Equation 21:

$$\begin{array}{l} L_{\max}=max\left\{l_{i};i=1,2,\cdots \;,N_{l}\right\} \end{array}$$ (21)

where *N*_*l*_ is the number of diagonal lines excluding the main diagonal.

(5) Diagonal Line Entropy (ENTR) is the Shannon entropy based on the diagonal line length distribution in the recurrence plot, reflecting the degree of uncertainty in the diagonal structure as shown in Equation 22.

$$\begin{array}{l} ENTR=-\overset{N}{\underset{l=l_{\min}}{\sum }}P\left(l\right)\ln\;P\left(l\right) \end{array}$$ (22)

(6) Laminarity (LAM) is the percentage of recurrence points that constitute vertical line structures, reflecting the degree of gradual change in system states and related to the system's intermittency as calculated in Equation 23.

$$\begin{array}{l} LAM=\frac{\sum_{\nu =\nu_{\min}}^{N}\nu P\left(\nu \right)}{\sum_{i=1}^{N}\sum_{j=1}^{N}R_{i,j}} \end{array}$$ (23)

where *v*_min_ is minimum vertical length, usually taken as 2, *P*(*v*) is probability distribution of vertical line segments of length *v*.

(7) Trapping Time (TT) is the mean of vertical structure lengths in the recurrence plot, reflecting the average duration the system remains in a specific state and related to the system's stickiness as shown in Equation 24.

$$\begin{array}{l} TT=\frac{\sum_{\nu =\nu_{\min}}^{N}\nu P^{\varepsilon }\left(\nu \right)}{\sum_{\nu =\nu_{\min}}^{N}P^{\varepsilon }\left(\nu \right)} \end{array}$$ (24)

(8) Maximum Vertical Line Length (*V*_max_) is the maximum length of vertical structures in the recurrence plot, with the calculation formula as shown in Equation 25:

$$\begin{array}{l} V_{\max}=max\left\{v_{i};i=1,2,\cdots \;,N_{v}\right\} \end{array}$$ (25)

where *v*_*i*_ is the set of all vertical line segments, *N*_v_–the number of vertical line segments.

(9) Recurrence Period Density Entropy (RPDE) is a normalized information entropy used to quantify the regularity of periodic structures in the system:

$$\begin{array}{l} RPDE=\frac{H\left(P\left(l\right)\right)}{H_{\max}}=\frac{-\sum_{l=l_{\min}}^{N}p\left(l\right)lnp\left(l\right)}{ln\left(L_{\max}-l_{\min}+1\right)} \end{array}$$ (26)

where *H*_max_ = ln(*L*_max_−*l*_min_+1) is the maximum entropy value under uniform distribution. The value of RPDE ranges from 0 to 1, RPDE = 0 indicates the system is completely periodic, while RPDE = 1 indicates the system is completely random.

**Figure 2:**
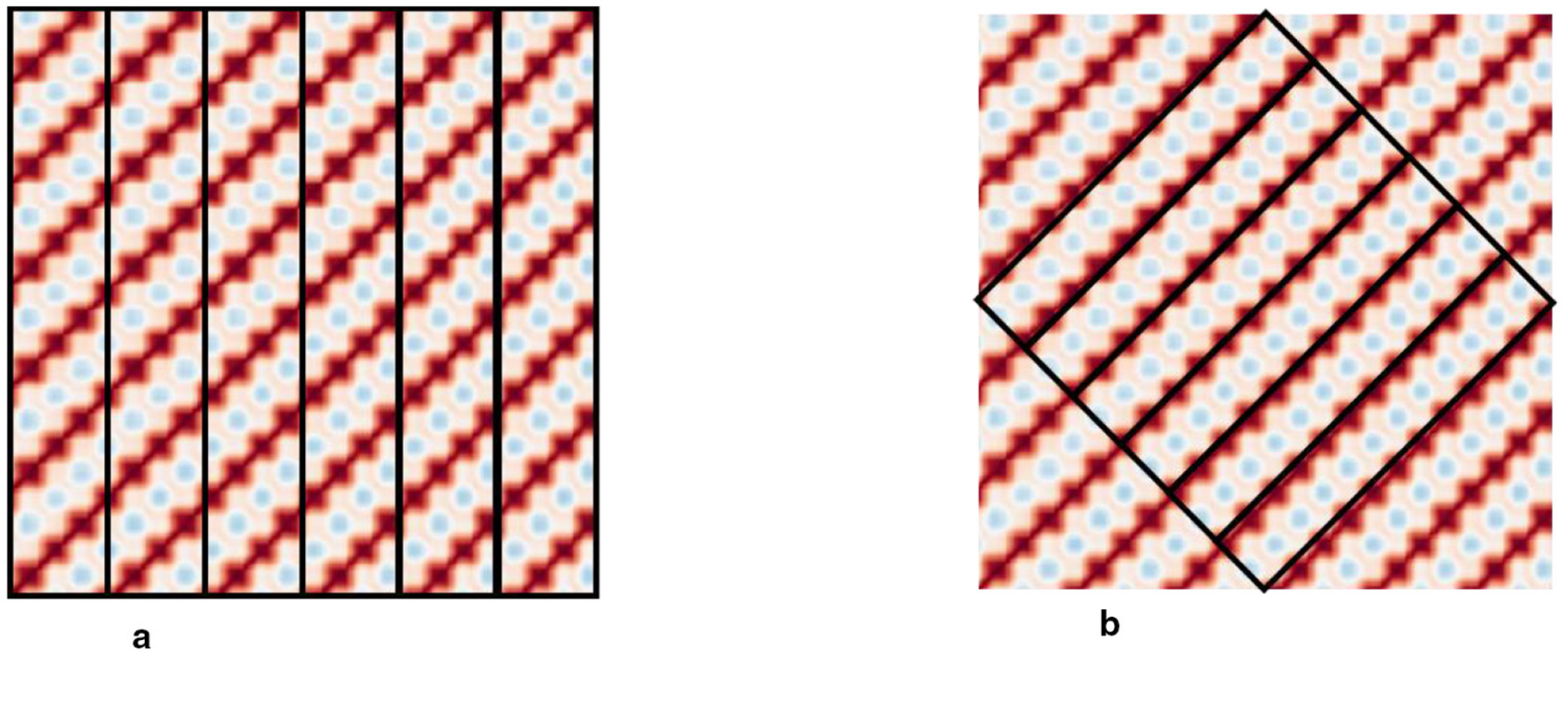
Blocks of non-thresholded recurrence plots. **(a)** Vertical block. **(b)** Diagonal block.

A total of nine entropy values are extracted in this study: approximate entropy, sample entropy, fuzzy entropy, Kolmogorov entropy, permutation entropy, modified conditional entropy, distribution entropy, slope entropy, and energy entropy. To extract local texture features of non-thresholded recurrence plots, the recurrence plot undergoes block processing. Blocks are created along the vertical and diagonal directions, and the mean and RMS values in the blocking direction are sequentially calculated to form one-dimensional sequences. Subsequently, the time-domain features and entropy features of the sequences are calculated to extract more detailed local texture features within each period. The recurrence plot has a size of 256 × 256 pixels. As showing Figure 2, for vertical block processing, the image is divided along the horizontal direction into strips with a width of 42 pixels and a stride of 42 pixels (non-overlapping). For diagonal block processing, a square is formed by connecting the midpoints of the four sides, and strips parallel to the diagonal direction are created with a width of 30 pixels and a stride of 30 pixels (also non-overlapping).

## 3 Experiments

The study adhered to the Declaration of Helsinki and was approved by the Xi'an Jiaotong University Health Science Center (n.2020-1330), with all subjects providing approved informed consent. A total of 61 patients with PD and 48 healthy subjects were recruited for the study, and the dataset is named as XJTU Gait. Experiments were conducted in the Department of Neurology at the First Affiliated Hospital of Xi'an Jiaotong University and on the Xing Qing Campus of Xi'an Jiaotong University. Basic demographic information including age, sex, height, and weight is shown in Table 2. The medication state is on, and the data we collected reflects patients' regular, ongoing medication regimen, which they were using prior to the study, without any modifications for research purposes. Healthy controls were recruited from community-dwelling older adults and matched to the PD group by age and sex. Exclusion criteria for healthy controls included any motor dysfunction (UPDRS-III score > 0), major comorbidities (e.g., hypertension, diabetes, cardiovascular disease, malignancy), or abnormal neurological examination findings.

**Table 2:** Basic information of participants (mean ± standard deviation).

Category	PD patients	Healthy subjects
Number (*n*)	61	48
Age (years)	63.6 (9.33)	55.2 (7.69)
Sex (male/female)	27/34	33/26
Height (cm)	166.02 (7.36)	162.15 (9.07)
Weight (kg)	64.51 (9.58)	66.11 (11.08)
Hoehn and Yahr stage	2.2	/

All participants were instructed to wear the gait-acquisition device developed (Xie et al., 2023; Zhao et al., 2022) for this project and completed gait tests. Prior to gait assessment, PD patients were evaluated by clinical specialists using standardized PD rating scales. The experimental procedures strictly followed the study protocol: (1) Physicians completed the PD patient case report form, basic demographic information, neurological examinations, and the MDS-UPDRS assessment. (2) For the gait-testing experiment, participants were required to wear the gait-acquisition device and perform normal walking, with each task repeated three times. Each participant completed 3 repetitions of walking. The preprocessing implemented in this work includes removal of initial and terminal effects, mean normalization, gait-cycle extraction, outlier elimination, and window segmentation in our previous study (Zhao et al., 2021).

## 4 Results

### 4.1 Recurrence plots

**Figure 3:**
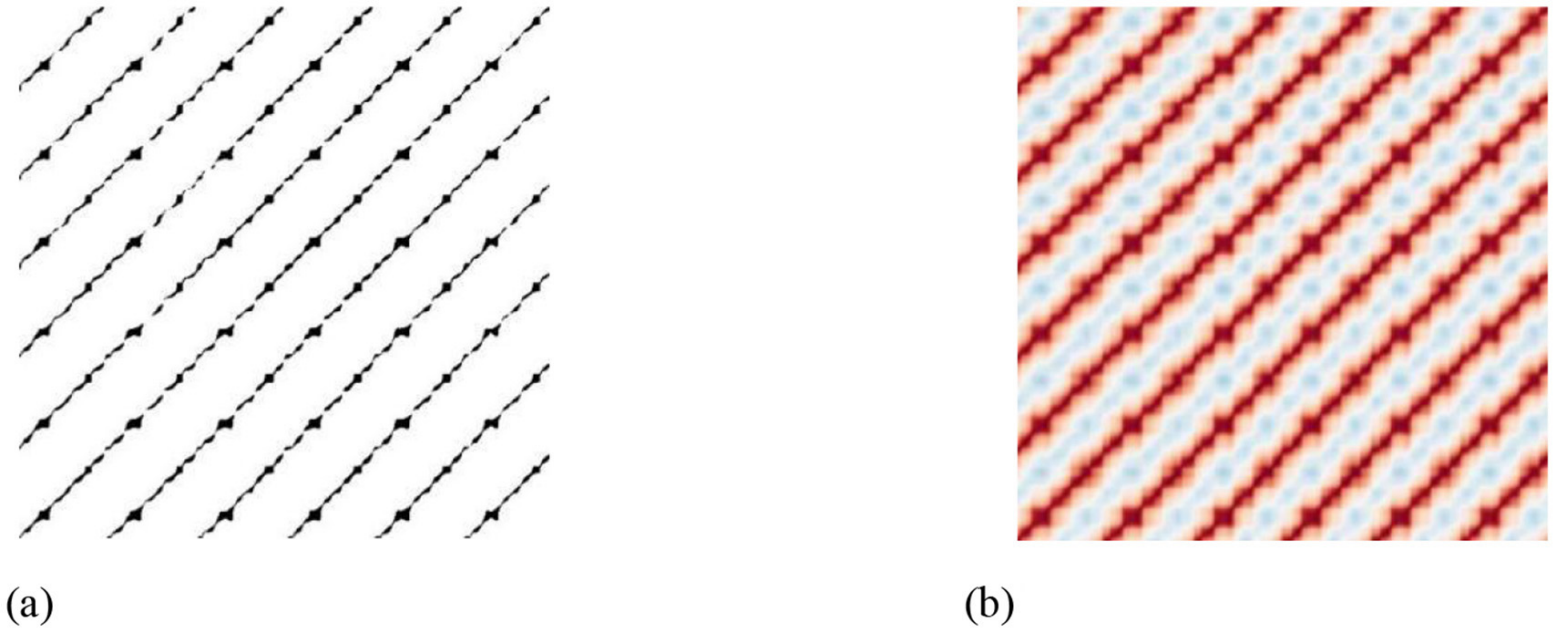
Diagrams of thresholded and non-thresholded recurrence plots. **(a)** Thresholded recurrence plot. **(b)** Non-thresholded recurrence plot.

Recurrence plots are shown in Figure 3, comparing thresholded and non-thresholded recurrence plots generated from plantar pressure signals, it is evident that the non-thresholded recurrence plot method can preserve much richer original information.

### 4.2 Recurrence quantification features

**Figure 4:**
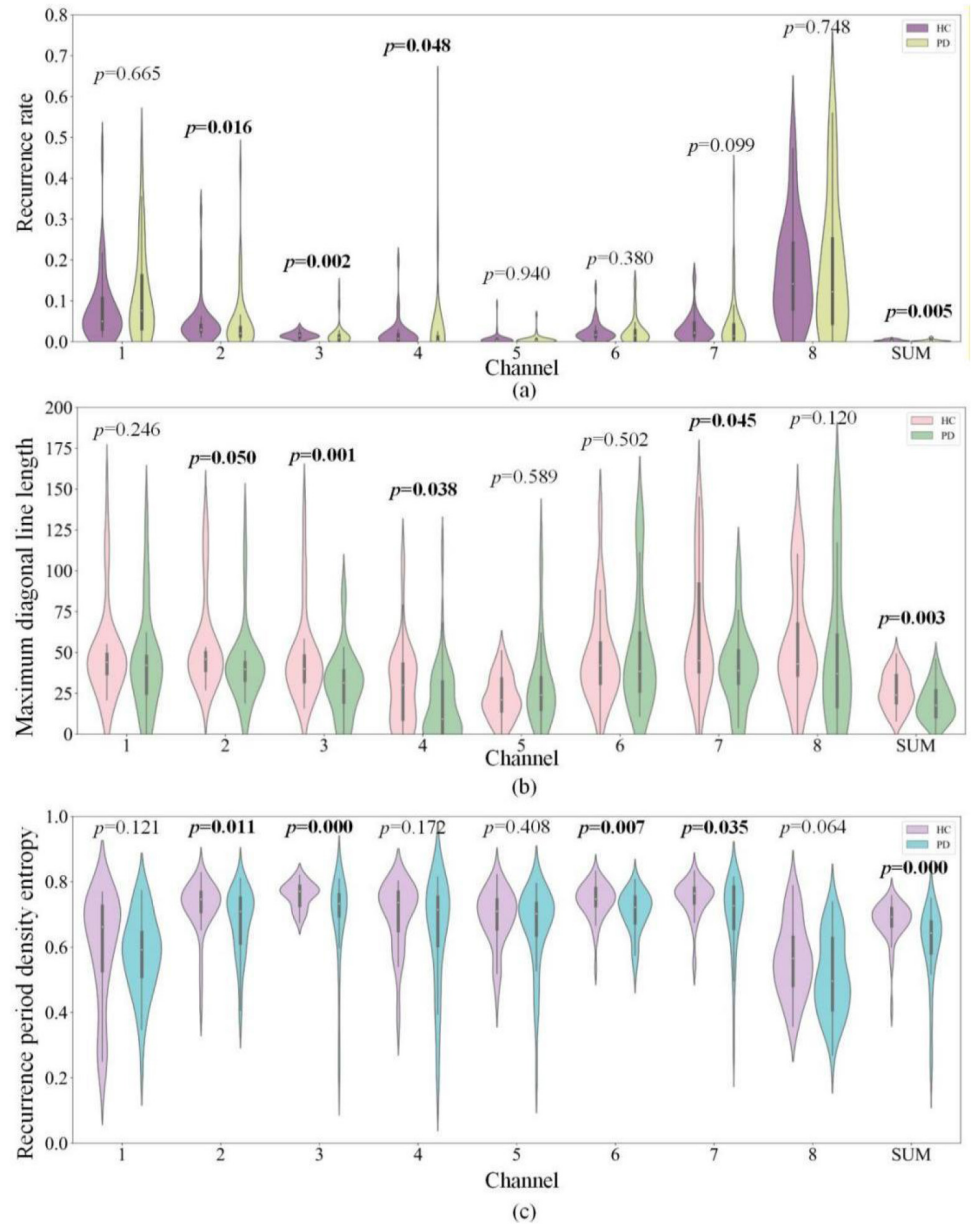
Violin plots of recurrence quantification analysis features for PD patients and healthy subjects. **(a)** Recurrence rate; **(b)** Maximum diagonal line length; **(c)** Recurrence period density entropy.

Due to space limitations, three feature sets that have more significant channels are selected for presentation: recurrence rate, maximum diagonal line length, and recurrence period density entropy of thresholded cross recurrence plots for each channel. The violin plots of feature distributions with the *p*-values are shown in Figure 4. As can be seen from the figures, PD patients show significantly lower values than healthy subjects in: recurrence rate of cross recurrence plots for Channels 3, 4, and Sum; maximum diagonal line length of cross recurrence plots for Channels 3, 4, 7, and Sum; and recurrence period density entropy of cross recurrence plots for Channels 3, 6, 7, and Sum. Other features in the figures show similar distributions and cannot distinguish between PD and HC, and no features of PD patients are significantly higher than those of healthy subjects. The recurrence quantification features of Channel 3 show significant differences, while the recurrence quantification features of Channels 2, 5, and 8 cannot effectively distinguish between PD patients and healthy subjects.

### 4.3 Recurrence plot entropy features

**Figure 5:**
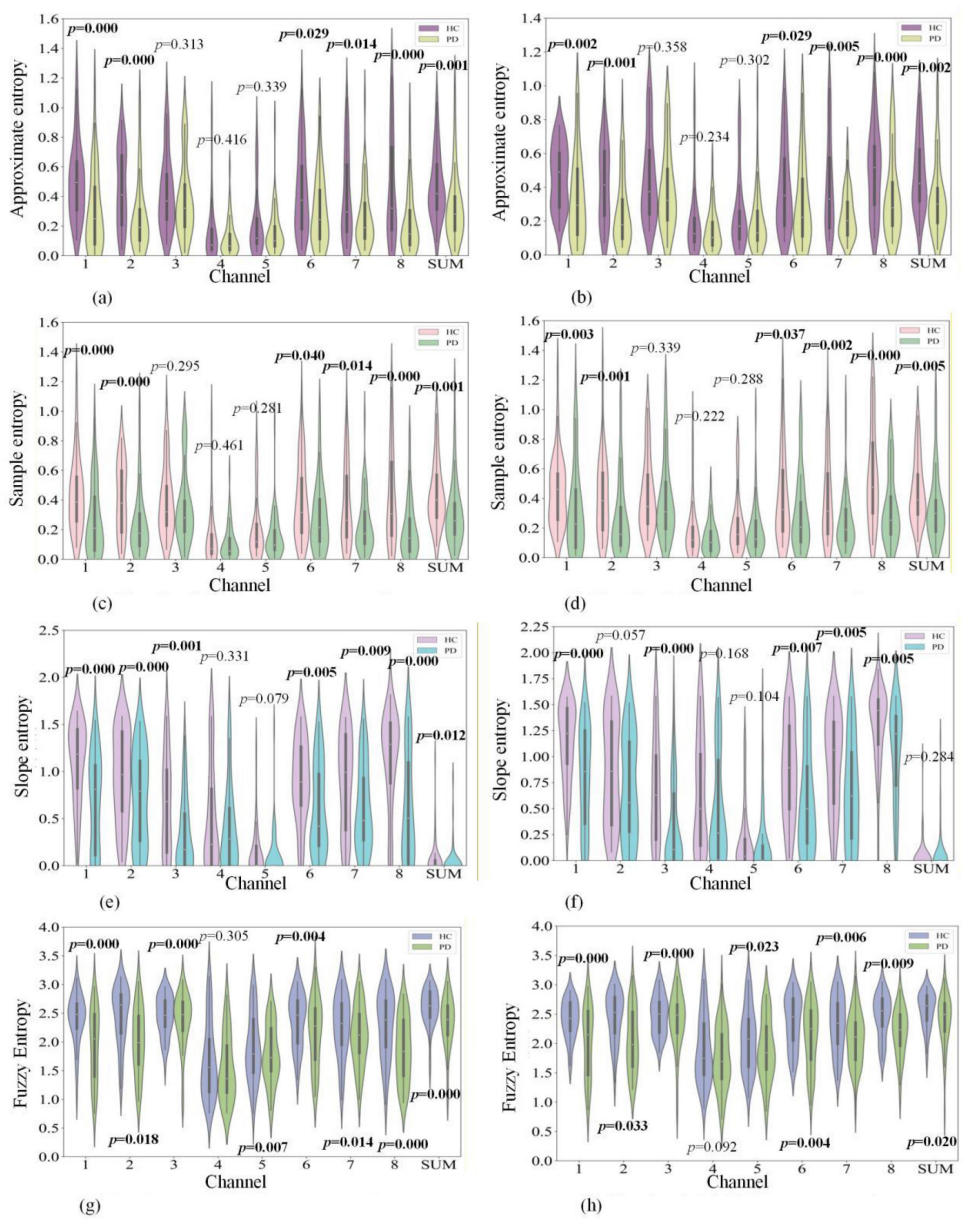
Violin plots of cross and joint recurrence plot entropy features for PD patients and healthy subjects. **(a)** Non-thresholded cross recurrence plots. **(b)** Non-thresholded joint recurrence plots. **(c)** Non-thresholded cross recurrence plots. **(d)** Non-thresholded joint recurrence plots. **(e)** Non-thresholded cross recurrence plots. **(f)** Non-thresholded joint recurrence plots. **(g)** Non-thresholded cross recurrence plots. **(h)** Non-thresholded joint recurrence plots.

Approximate entropy, sample entropy, slope entropy, and energy entropy of non-thresholded cross recurrence plots and joint recurrence plots for each channel. The violin plots of feature distributions and their corresponding *p*-values are shown in Figure 5.

It can be observed that most entropy features of Channel 2 in non-thresholded cross recurrence plots show significant differences, while the entropy features of Channels 4 and 5 cannot effectively distinguish between PD patients and healthy subjects. Furthermore, the fuzzy entropy feature of non-thresholded cross recurrence plots shows significant differences across most channels, while the distribution entropy feature cannot effectively distinguish between PD patients and healthy subjects across most channels. For non-thresholded joint recurrence plots, most entropy features of Channel 8 show significant differences, while the entropy features of Channel 4 cannot effectively distinguish between PD patients and healthy subjects. Moreover, it was found that the fuzzy entropy feature of non-thresholded joint recurrence plots shows significant differences across most channels. PD patients show significantly lower values than healthy subjects in approximate entropy and sample entropy of cross and joint recurrence plots for Channels 1, 2, 6, 7, 8, and Sum; slope entropy of cross recurrence plots for Channels 1, 2, 3, 6, 7, 8, and Sum, and joint recurrence plots for Channels 1, 3, 6, 7, and 8; and energy entropy of cross recurrence plots for Channels 1, 2, 8, and Sum, and joint recurrence plots for Channels 1, 2, 6, 7, 8, and Sum.

### 4.4 Feature comparison

To systematically evaluate the effectiveness of the extracted features, statistical difference analysis of features were conducted. The significance level was measured by *p*-values, evaluated through their distribution and minimum values. When *p*-value < 0.05, the feature difference is considered statistically significant. The distribution of features with *p* < 0.05 was statistically analyzed to assess the discriminative power of various feature types, various types of recurrence plots, and recurrence plots from various channels, and to identify objective biological markers that can characterize PD.

#### 4.4.1 Comparison of different feature types

A comparative analysis of the effectiveness of different feature types was conducted, with their feature significance levels (characterized by *p*-values) shown in Table 3. From the results in the table, it can be seen that the minimum *p*-value of recurrence plot entropy features is 4.41 × 10^−09^, the lowest among the four feature categories, with the largest number of features within the same order of magnitude, demonstrating the superiority of recurrence plot entropy features. In particular, the minimum *p*-value of recurrence plot entropy features is far lower than that of recurrence quantification features, and the number of features with *p*-values in the same order of magnitude for recurrence plot entropy features is also far greater than that for recurrence quantification features. Clearly, recurrence plot entropy features can better characterize the differences between PD patients and HCs.

**Table 3:** Distribution of different feature types.

Feature type	Min *p*-value	Features with *p* < 0.05	Features with *p* < 0.001	Features with *p* < 1.0E−05	Features with *p* < 1.0E−07
Pressure features	3.76E-08	93	46	15	4
Temporal features	5.57E−07	56	16	6	0
Recurrence quantification features	8.23E−06	60	8	2	0
Recurrence plot entropy features	4.41E−09	557	186	47	12

#### 4.4.2 Comparison of different types of recurrence plot features

The effective features of cross recurrence plots and joint recurrence plots across all channels were compared. The *p*-value distribution of features is shown in Table 4 below. As can be seen from the table, the feature differences of cross recurrence plots and joint recurrence plots involving bilateral foot signals are both stronger than basic recurrence plots constructed from any single foot signal, reflected in smaller minimum *p*-values and a greater number of features with *p*-values in the same order of magnitude. Comparing cross recurrence plots with joint recurrence plots, cross recurrence plot features also lead joint recurrence plots in all aspects. Clearly, cross recurrence plot entropy features can better characterize the differences between PD patients and healthy subjects.

**Table 4:** Feature distribution of cross recurrence plots and joint recurrence plots.

Recurrence plot	Min *p*-value	Features with *p* < 0.05	Features with *p* < 0.001	Features with *p* < 1.0E−05	Features with *p* < 1.0E−07
Left foot	9.64E−08	84	22	5	1
Right foot	3.90E−08	122	43	11	2
Cross	4.41E−09	216	77	20	6
Joint	3.75E−08	195	52	13	3

#### 4.4.3 Comparison of recurrence plots from individual channels

The *p*-value distribution of cross recurrence plot features for each channel is shown in Table 5. From the results in the table, it can be seen that Channels 1 and 8 have more features with small *p*-values, while the *p*-values of features from Channels 4, 5, 6, and 7 are all relatively large, all exceeding 0.001. Therefore, it is determined that signals from Channels 1 and 8 better reflect the differences between PD patients and healthy subjects, i.e., cross recurrence plot features of pressure signals from the heel and toe positions have greater specificity.

**Table 5:** Feature distribution of recurrence plots from individual channels.

Channel	Min *p*-value	Features with *p* < 0.05	Features with *p* < 0.001	Features with *p* < 1.0E−05	Features with *p* < 1.0E−07
1	4.41E−09	45	22	8	4
2	9.12E−07	36	14	3	0
3	4.46E−06	23	9	2	0
4	9.53E−03	7	0	0	0
5	2.19E−02	3	0	0	0
6	2.54E−03	15	0	0	0
7	1.73E−03	16	0	0	0
8	1.30E−08	48	24	7	2
Sum	3.16E−05	23	8	0	0

### 4.5 Feature diagnosis results analysis

#### 4.5.1 Feature selection

Mann–Whitney *U*-test and Pearson correlation coefficient analysis were employs to complete feature selection. The Mann-Whitney U test is used to test whether two groups of data come from continuous distribution samples with equal medians and whether there are significant differences between them. The *p*-value is the probability of observing a test statistic as extreme or more extreme than the observed value under the null hypothesis. A *p*-value less than 0.05 indicates a significant difference between the two groups of data. Pearson correlation coefficient analysis is used to remove redundant features. It returns the pairwise correlation coefficient matrix between each column in the input matrices, calculating the correlation coefficients between features. For highly correlated features, elimination operations are performed, retaining only one representative feature set. Temporal and pressure features (Zhao et al., 2021), cross and joint recurrence plot features from eight channels of both feet were extracted, and features with *p*-values lower than 0.05 or importance ratios higher than 0.03 were retained.

#### 4.5.2 Feature diagnosis

**Figure 6:**
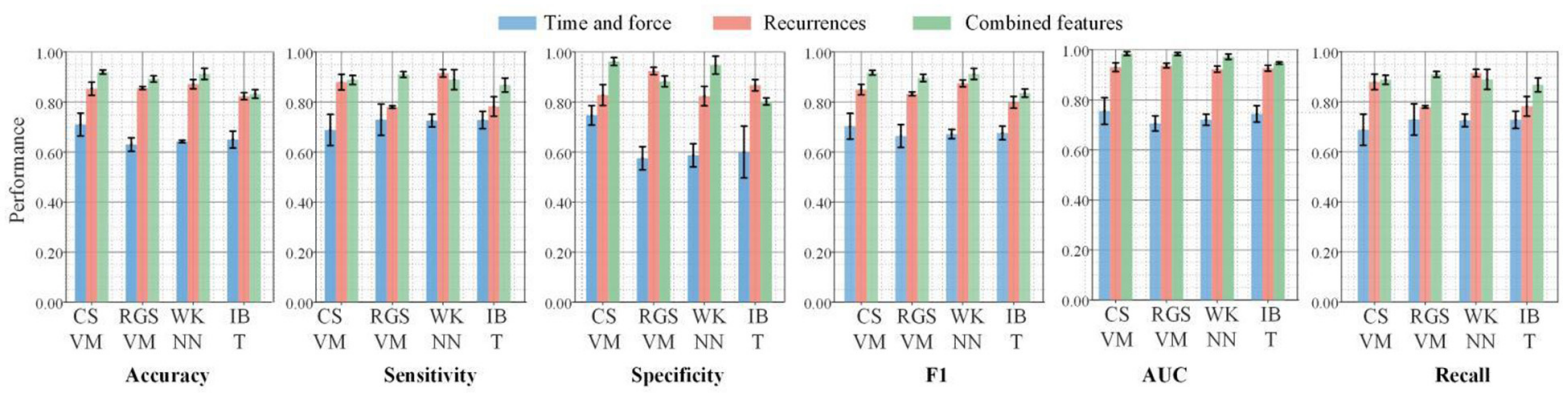
Performances of features for different diagnosis models.

The selected features were applied to classify PD patients and healthy subjects. Training employed integrated classifier models in MATLAB, including Cubic Support Vector Machine (CSVM), Rough Gaussian Support Vector Machine (RGSVM), Weighted K-Nearest Neighbors (WKNN), and Integrated Bagging Tree (IBT). The 5-fold-cross-validation was used to train and test the models, and it was repeated 4 times. Using 34 temporal and pressure features, 252 recurrence plot features, and 286 integrated features as model inputs respectively, the classification accuracy results for each model are shown in Figure 6. Six evaluation metrics were evaluated, including accuracy, sensitivity, specificity, *F*1-score, AUC, and recall. The bars represent the mean values, and the error bars indicate the standard deviation obtained from repeated cross-validation.

Using time and force features, the classification accuracy ranged from approximately 62% to 73% across different classifiers. The sensitivity and *F*1-score were relatively lower, while the specificity reached approximately 90% in some models, indicating that the classifiers were more effective at identifying negative samples than positive ones when only time and force features were used.

When recurrence features were applied, the overall performance improved. The accuracy increased to approximately 75%−86%, while both sensitivity and recall exceeded 80% for most classifiers. In addition, the AUC values improved to approximately 90%−94%, indicating a stronger discriminative capability compared with the time and force feature set. The best performance was achieved using the combined feature set. Specifically, the WKN classifier achieved the highest performance, with an accuracy of 91.93% ± 1.41%, sensitivity of 89.25% ± 2.27%, specificity of 95.05% ± 0.72%, F1-score of 91.85% ± 1.46%, and AUC of 97.87% ± 0.72%. These results demonstrate that integrating time and force features with recurrence features significantly enhances the classification performance. Overall, the combined feature set consistently outperformed the single feature sets across all evaluation metrics, suggesting that combination of different kinds of features provides complementary information and improves model robustness and discriminative ability.

## 5 Discussions

The observed reduction in recurrence plot entropy and recursive quantization features in patients with Parkinson's disease may be associated with the underlying neuropathological mechanisms of the disease. PD is characterized by the degeneration of dopaminergic neurons in the Substantia nigra pars compacta, which leads to dopamine depletion in the Basal ganglia and disrupts the normal functioning of the basal ganglia–cortical motor circuit. This dysfunction impairs the automatic control of locomotion and reduces the adaptability and variability of motor output. Consequently, gait patterns in PD patients tend to become more rigid, stereotyped, and predictable due to symptoms such as bradykinesia and rigidity. From a nonlinear dynamics perspective, these pathological changes reduce the complexity and dynamic variability of gait signals, leading to more regular and constrained trajectories in the reconstructed phase space. As a result, measures reflecting signal complexity and state diversity, such as recurrence plot entropy and recursive quantization features, are significantly lower in PD patients compared with healthy controls.

The superior performance of heel and toe channels can be attributed to the specific motor deficits characteristic of PD. Heel strike is a critical component of gait initiation and is often reduced or absent in PD patients due to bradykinesia and rigidity, leading to a flat-footed landing. Similarly, toe-off, which requires propulsive force from the forefoot, is typically diminished, resulting in shorter stride lengths and a shuffling gait (Zhao et al., 2026). The RQA and entropy features derived from these regions are therefore highly sensitive to these specific motor impairments. In contrast, mid-foot signals may be more influenced by individual anatomical variations and less directly by PD-related pathophysiology, explaining their lower specificity in our models.

Comparisons to the state of art are shown in Table 6. It can be observed that the WKNN method based our features achieved 90.99% accuracy in cross-subject validation on the XJTU Gait dataset, outperforming prior deep-learning approaches in generalizability. By combining temporal, force, and recurrence features, it provides richer gait representation than methods relying solely on CNNs or feature-less models, while maintaining competitive accuracy. Although some approaches report slightly higher accuracy in non-cross-subject settings, they lack cross-subject robustness, highlighting the balanced performance of this study.

**Table 6:** Comparisons to the state of art.

References	Data	Methods	Features	Accuracy/%	Validation
Navita et al. (2025)	Public dataset ≪gait in Parkinson's disease≫	Hypertuned random forest tree	Time, frequency, spatial, and temporal features	97.5 (2.1)	Non-cross subject
Ma et al. (2023)		CNN	Statistic features	98.41	Non-cross subject
Naimi et al. (2024)		Hybrid ConvNet-transformer	No features	87.89	Cross subject
Zhao et al. (2025)	Part of XJTU gait	Federated transfer multi-channel CNN	No features	83.94 (9.15)	Cross subject
This study	XJTU gait	WKNN	Combined temporal features, force features, and recurrence features	90.99 (1.38)	Cross subject

Several limitations should be acknowledged. First, the model was developed using a specific cohort and the self-design system, which may limit its generalizability to other populations or gait-acquisition devices. Second, the relatively small sample size and the fixed 16-sensor configuration may introduce a risk of overfitting, although cross-validation was applied to reduce this risk. Finally, the model was validated only using hold-out data from a single-center dataset, and external validation with independent, multi-center cohorts is needed to confirm its robustness and potential clinical applicability in the future.

## 6 Conclusions

To detect PD automatically, recurrence plot algorithms were introduced, including thresholded recurrence plots, non-thresholded recurrence plots, basic recurrence plots, cross recurrence plots, and joint recurrence plots. Texture analysis was performed on recurrence plots generated from gait signals to identify texture differences between PD patients and HC. The recurrence plots of gait signals from PD patients exhibited lower complexity. Recurrence quantification features and recurrence plot entropy features were extracted from gait signals, and feature distribution differences were analyzed through violin plots. The results demonstrated that the extracted features can effectively distinguish between PD patients and HC. Among the four feature categories, recurrence plot entropy features showed the most excellent performance. Cross recurrence plots outperformed joint recurrence plots and basic recurrence plots. Finally, preliminary diagnosis of PD was conducted based on integrated features, achieving a maximum diagnostic accuracy of 92.71%.

## Data Availability

The raw data supporting the conclusions of this article will be made available by the authors, without undue reservation.
